# Voltage-Dependent Sarcolemmal Ion Channel Abnormalities in the Dystrophin-Deficient Heart

**DOI:** 10.3390/ijms19113296

**Published:** 2018-10-23

**Authors:** Xaver Koenig, Janine Ebner, Karlheinz Hilber

**Affiliations:** Department of Neurophysiology and—Pharmacology, Center for Physiology and Pharmacology, Medical University of Vienna, Schwarzspanierstraße 17, 1090 Vienna, Austria; janine.ebner@meduniwien.ac.at

**Keywords:** animal models, calcium channels, cardiac ion channel abnormalities, dystrophic cardiomyopathy, dystrophin deficiency, dystrophinopathies, potassium channels, sodium channels

## Abstract

Mutations in the gene encoding for the intracellular protein dystrophin cause severe forms of muscular dystrophy. These so-called dystrophinopathies are characterized by skeletal muscle weakness and degeneration. Dystrophin deficiency also gives rise to considerable complications in the heart, including cardiomyopathy development and arrhythmias. The current understanding of the pathomechanisms in the dystrophic heart is limited, but there is growing evidence that dysfunctional voltage-dependent ion channels in dystrophin-deficient cardiomyocytes play a significant role. Herein, we summarize the current knowledge about abnormalities in voltage-dependent sarcolemmal ion channel properties in the dystrophic heart, and discuss the potentially underlying mechanisms, as well as their pathophysiological relevance.

## 1. Introduction

Dystrophin deficiency causes diseases, the so-called “dystrophinopathies”, with progressive muscle weakness and cycles of muscle necrosis and regeneration representing the pathophysiological hallmarks [1]. Duchenne muscular dystrophy (DMD) is the most common and devastating form. Boys with DMD (approximately 1 in 3500 are affected) usually show motor difficulties by six years of age. Thereafter, muscle weakness progresses and leaves patients wheelchair-bound by their teens. Death usually occurs before the patients become forty. The gene defect for DMD was mapped to an X chromosome gene that encodes for the intracellular protein dystrophin. This protein is part of a multimeric protein complex, the so-called dystrophin-associated protein complex (DAPC) [2]. Via DAPC, dystrophin provides a link between the intracellular microfilament network of actin and the extracellular matrix [2,3]. In the absence of this physical link, as in the case of dystrophin deficiency, the muscle fibers show an increased sensitivity to mechanical stress [4] and become susceptible to degeneration. While DMD is characterized by the absence of dystrophin, mutations that result in the retention of a partly functional dystrophin gene product are characteristic for the less severe dystrophinopathy type, called Becker muscular dystrophy (BMD) (incidence: at least 1 in 18,450 male live births [5]). Like DMD, BMD is characterized by progressive skeletal muscle weakness, but shows a more heterogeneous clinical picture and has a milder course [6]. BMD patients may live until the fifth or sixth decade of life [7]. Although the detailed mechanism(s) responsible for muscle fiber degeneration in the course of dystrophinopathy progression are not completely understood, there is general agreement on the involvement of abnormally enhanced levels of intracellular Ca^2+^ in dystrophic cells [8,9,10].

Besides skeletal muscle degeneration, the dystrophinopathies are frequently associated with cardiovascular complications, including the development of a dilated cardiomyopathy and cardiac arrhythmias [6,9,11,12,13]. Interestingly, in contrast to the normally more pronounced skeletal muscle phenotype observed in DMD compared with BMD, the cardiac disease phenotype is often considered rather similar in both types of dystrophinopathies [14,15,16], or even more severe in BMD patients (e.g., [17,18]). This entails a difference in the main clinical feature associated with the two diseases, namely: the skeletal muscle phenotype is typically prevailing in DMD, whereas in BMD, the cardiac phenotype can be the predominant pathology [6,7,19,20]. For BMD, symptoms usually occur in the third decade of life. About one third of the patients develop a dilated cardiomyopathy with accompanying heart failure. In addition, arrhythmias occur [6,21], and the cardiac involvement significantly contributes to the morbidity and mortality observed [6,17,20]. There is no obvious correlation between cardiac involvement and the severity of skeletal muscle pathology in BMD patients [6]. Notably, female BMD and DMD carriers, who are mostly free of skeletal muscle symptoms, are also prone to cardiomyopathy development [22,23]. As the specific mechanisms resulting in cardiac complications in the course of dystrophinopathy progression are incompletely understood, current therapy is not targeted, but relies on approaches that are considered standard for dilated cardiomyopathy [20,24]. Concerning cardiac arrhythmia management, there are no effective treatments to prevent lethal ventricular tachyarrhythmias, because of a lack of understanding about the underlying mechanisms (e.g., [25]). Consequently, there is an urgent need for boosting fundamental research in this field as a basis for the future development of targeted therapeutic strategies in order to specifically manage the cardiac complications associated with the dystrophinopathies [10,26].

In recent years, it has become apparent that the functional properties of voltage-dependent sarcolemmal ion channels are significantly disturbed in dystrophin-deficient cardiomyocytes, and relevance for the pathophysiology in the dystrophic heart has been suggested. These ion channel abnormalities are likely connected with the fact that several channels directly interact with protein members of the DAPC (e.g., [27,28,29]), and can thus be considered DAPC members themselves. Alterations in the composition of this multiprotein complex (e.g., due to dystrophin deficiency) will therefore impact the associated channels. Importantly, in this context, ion channels (and their regulators) in dystrophin-deficient cardiomyocytes represent potential new therapeutic targets for the management of dystrophic cardiomyopathy in patients. In the present article, we review the current knowledge about the abnormalities in the voltage-dependent sarcolemmal sodium, calcium, and potassium channels in the dystrophic heart. Although important, this topic has so far not received much attention. Other ion channels with potential relevance in dystrophic cardiomyopathy, such as for example transient receptor potential (TRP) channels [30,31] or the K_(ATP)_ channel [32], are not considered herein.

## 2. Dystrophic Cardiac Ion Channel Abnormalities: Evidence from Animal Model Studies

### 2.1. Sodium Channels

**Figure 1:**
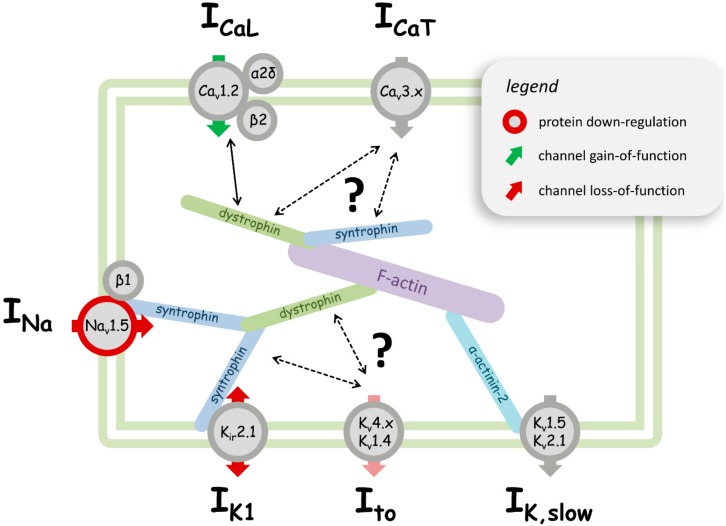
Cartoon summarizing the effects of dystrophin deficiency on voltage-dependent sarcolemmal ion channel expression and function in a ventricular cardiomyocyte. In healthy cardiomyocytes, protein members of the dystrophin-associated protein complex (DAPC), such as dystrophin and syntrophin, interact with ion channels to ensure their proper cellular expression and function. Among those interactions, syntrophin binding to Na_v_1.5 and K_ir_2.1 is well-established (see manuscript text). For potential other interactions (indicated by arrows), the evidence is less strong. In case of dystrophin deficiency, the DAPC interactions with ion channels are disturbed, which results in alterations in the channel properties. A red circle around a particular ion channel represents the down-regulation of the expression of the respective protein, while a grey circle indicates that dystrophin deficiency does not (or is not known to) alter a channel’s or a channel subunit’s expression. Red and green arrows represent channel loss-of-function and gain-of-function, respectively. The light red arrow indicates potential channel loss-of-function, whereby the literature evidence is controversial. A grey arrow indicates unaltered channel function, or that the potential effects of dystrophin deficiency are unknown. The physiologically prevailing direction of current flow during an action potential through a particular ion channel (inward and/or outward) can be derived from the respective arrow head position(s). I_CaL_—L-type Ca^2+^ current; I_CaT_—T-type Ca^2+^ current; I_Na_—Na^2+^ current; I_K1_—inward rectifier K^+^ current; I_to_—transient outward K^+^ current; I_K,slow_—ultra-rapid delayed rectifying and slowly inactivating K^+^ current. The channel or channel subunit names in the circles are given according to IUPHAR nomenclature.

In adult ventricular cardiomyocytes, voltage-dependent sodium (Na^+^) channels are responsible for the rapid depolarization during the upstroke phase of the action potential (AP), and Na_v_1.5 is the dominant channel isoform expressed. We [33] and others [34,35,36] have previously used various mouse models of DMD/BMD (mdx—dystrophin-deficient, classical dystrophinopathy mouse model [37]; mdx^5cv^—mdx strain with a different point mutation [38]; mdx-utr—both dystrophin- and utrophin-deficient, more severe disease phenotype [39,40]) to study the properties of Na^+^ channels in dystrophic cardiomyocytes. These studies have consistently revealed that Na^+^ currents (i.e., Na^+^ current densities) are significantly reduced in the cardiomyocytes derived from the ventricles of adult dystrophin-deficient mice when compared to wild type [33,34,35,36]. This suggested that dystrophin deficiency reduces the number of functional Na^+^ channels in the cardiomyocyte membrane, which was supported by decreased Na_v_1.5 protein levels in the heart samples of dystrophic mice [34,35,36,41] (Figure 1, Table 1).

Because Na_v_1.5 interacts with dystrophin [34] and other protein members of the DAPC, the syntrophins [28,29,34], it is conceivable that the disturbance of these interactions, in the case of dystrophin deficiency, impairs Na_v_1.5 expression and localization. The interaction with syntrophin normally occurs via the binding of the last three C-terminal residues of Na_v_1.5 (Ser-Ile-Val) to syntrophin’s PDZ domain [54]. A detailed analysis of the Na_v_1.5 expression and localization in healthy mouse ventricular cardiomyocytes revealed that at least two pools of channels coexist in the membrane, as follows: one targeted at the lateral membranes by the DAPC via syntrophin and dystrophin, and one targeted at the intercalated disks by synapse associated protein, SAP97 [55]. These authors further reported a selective loss of Na_v_1.5 at the lateral membranes, but not at the intercalated disks, in dystrophin-deficient (mdx) cardiomyocytes. Obviously, functional dystrophin is essential for a proper expression of Na_v_1.5 at the lateral membranes of ventricular cardiomyocytes, and the loss of this protein leads to impaired channel expression and, consequently, Na^+^ channel loss-of-function. Moreover, it was suggested that the reduced expression of Na_v_1.5 in dystrophin-deficient cardiomyocytes is dependent on proteasomal degradation [36].

Besides markedly reduced Na^+^ current (I_Na_) densities and Na_v_1.5 protein levels in ventricular cardiomyocytes derived from adult dystrophin-deficient mice, the gating properties of the expressed Na^+^ channels are normal or slightly impaired at most. Thus, the voltage-dependencies of the activation and inactivation in dystrophic ventricular cardiomyocytes were found to be similar to those of wild type myocytes [33,34,36]. Only the authors of [35] reported slight shifts in both the activation and steady-state inactivation curves towards more positive potentials in dystrophic cardiomyocytes. We therefore conclude that a reduction in the lateral membrane expression represents the major Na^+^ channel abnormality in dystrophin-deficient ventricular cardiomyocytes.

Reduced I_Na_ in dystrophin-deficient ventricular cardiomyocytes should affect their action potential (AP). Indeed, we found a significant decrease in the AP upstroke velocity and a diminished AP amplitude in dystrophic cardiomyocytes [33]. The described AP abnormalities in dystrophic myocytes accord with reduced I_Na_, and their relevance for the dystrophic heart was studied by comparing the electrocardiograms (ECGs) recorded from adult wild type and dystrophin-deficient mice. Here, consistent with the reduced Na^+^ channel availability and a slower AP upstroke in dystrophic ventricular cardiomyocytes, several groups found significantly prolonged QRS intervals in the ECGs of dystrophic, compared with wild type mice [34,41,56]. This suggests that ventricular conduction is slowed in dystrophic animals. It should be noted that, besides reduced I_Na_, other abnormal properties of cardiomyocytes could also affect ventricular conduction in the dystrophic heart. For example, a differential expression of connexin isoforms compared to wild type [41] would have a direct impact on conduction.

### 2.2. Calcium Channels

During the plateau phase of the ventricular AP, a calcium (Ca^2+^) influx through Ca_v_1.2 L-type Ca^2+^ channels into the cytosol elicits a Ca^2+^-induced Ca^2+^ release from the sarcoplasmic reticulum (SR), which finally triggers contraction. Comparison of the functional properties of Ca_v_1.2 in ventricular cardiomyocytes derived from wild type and dystrophic mice revealed two major differences, both of which reflecting a gain-of-function, namely: (i) enhanced Ca^2+^ current (I_Ca_) densities [42,49]; and (ii) reduced channel inactivation [27,33,44,49,50,51] in dystrophic compared to wild type myocytes. In spite of some differences among the results from the above-named studies, all of the authors consistently reported a channel gain-of-function in dystrophic cells (Figure 1, Table 1). This is in agreement with the increased in-vivo left ventricular Ca^2+^ influx in the mdx mouse detected by manganese-enhanced cardiovascular magnetic resonance imaging [57]. On the other hand, two reports of normal Ca_v_1.2 properties in dystrophic ventricular cardiomyocytes do exist [47,48]. To some extent, the inconsistencies in the findings between various groups may be explained by the use of mouse populations of different ages. Accordingly, the authors of [42] reported that the Ca^2+^ channel abnormalities in dystrophic cardiomyocytes were dependent on the age of the animals from which the cells had been derived.

Ca_v_1.2 inactivates in a Ca^2+^-, as well as in a voltage-dependent manner. To test if both of these modes of inactivation were impaired in dystrophic cardiomyocytes, we measured currents through Ca^2+^ channels using either Ca^2+^ or barium (Ba^2+^) as the charge carrier [49]. While I_Ca_ shows Ca^2+^-dependent inactivation as the predominant form, Ba^2+^ currents exclusively exhibit voltage-dependent inactivation. We found reduced inactivation both in the Ca^2+^ and Ba^2+^ currents of dystrophic ventricular cardiomyocytes [49]. This implied that dystrophin deficiency impairs both Ca^2+^- and voltage-dependent inactivation.

All of the above cited studies have used hearts from either neonatal or young adult dystrophic mice as a cell source. Recently, we have studied I_Ca_ in ventricular cardiomyocytes derived from aged (>1 year of age) mdx mice. To our surprise, we found that the functional properties of Ca_v_1.2 were similar in cardiomyocytes from mdx and wild type mice at this age [52]. This implied that the dystrophin regulation of Ca_v_1.2 in the heart is lost during aging. A significant loss of dystrophin protein in the senescent murine heart [58] may help to explain this unexpected finding.

In contrast to L-type Ca_v_1.2, low-voltage activated T-type Ca^2+^ channels are only slightly expressed in cardiac ventricles from healthy adult mice, but the ventricular re-expression of T-type channels has been reported in heart failure [59,60]. In the course of our previous work using 15–25-week-old mdx mice, with a focus on the L-type Ca^2+^ channel, we could only detect minute T-type I_Ca_ both in wild type and mdx ventricular cardiomyocytes, and we have not noticed considerable differences in the current density or voltage-dependence of channel activation between normal and dystrophic cells. The activation as well as inactivation kinetics of the T-type I_Ca_ also appeared to be similar. Tiny T-type I_Ca_ with similar properties was also found in wild type and mdx ventricular cardiomyocytes derived from aged (>1 year of age) mice [52]. We therefore believe that T-type I_Ca_ is neither considerably upregulated, nor dysregulated in dystrophic cardiomyocytes.

Studies that have provided evidence for an interaction of L-type Ca^2+^ channels with dystrophin in the heart [27] and in skeletal muscle [61] suggested that, similar to that shown for Na_v_1.5 (see above), the expression of L-type Ca^2+^ channels may be regulated by dystrophin. This, however, does not seem to be the case, because, first, Ca^2+^ channel main alpha1C- [34,42,43,44,49] and auxiliary- [42,45,49] subunit expression both at mRNA and protein level was found to be similar in wild type and dystrophic mouse hearts (also see Table 1A). Secondly, ON-gating charge values, a measure for the number of channels in the membrane, were similar in wild type and dystrophic cardiomyocytes [49]. Furthermore, the absence of dystrophin did not appear to affect the membrane localization of Ca_v_1.2 in cardiac tissue ([27]; own unpublished preliminary data).

If abnormal Ca_v_1.2, and channel subunit expression cannot account for the altered I_Ca_ properties (enhanced current density and reduced channel inactivation) in dystrophic cardiomyocytes, there must be one or several other causative mechanisms. It is, for example, conceivable that the lack of dystrophin impairs Ca_v_1.2 regulation. In this context, the authors of [42] tested the beta-adrenergic regulation of Ca_v_1.2 in wild type and mdx mice, and reported an increased basal phosphorylation of the Ca_v_1.2 alpha1C subunit, accompanied by enhanced cAMP-dependent protein kinase A (PKA) activity in the hearts from mdx mice. The PKA-dependent modulation of Ca_v_1.2 (by phosphorylation [62,63] and/or other mechanisms [64,65]) is thought to enhance the I_Ca_ amplitude, and also to affect the channel inactivation properties [64]. An increased baseline PKA activity, and consequentially enhanced basal phosphorylation of Ca_v_1.2, may provide an explanation for larger L-type I_Ca_ in mdx cardiomyocytes. The concept of an increased baseline PKA activity in dystrophic cardiomyocytes is supported by enhanced PKA phosphorylation of the ryanodine receptor (RYR_2_) associated with dystrophic cardiomyopathy in mdx mice [66]. On the other hand, it is in contrast to the increased I_Ca_ enhancement triggered by beta-adrenergic stimulation via isoprenaline in mdx compared to wild type cardiomyocytes [27]. Further studies are required to fully understand to what extent, and how exactly abnormal PKA regulation contributes to L-type Ca^2+^ channel gain-of-function in the dystrophic heart.

Besides abnormal PKA modulation, another potential source of I_Ca_ dysregulation in the dystrophic heart is nitric oxide synthase (NOS). In cardiomyocytes, nitric oxide (NO), synthesized by NO synthases, is believed to regulate the function of Ca_v_1.2 (e.g., [67,68]). Among the different NOS isoforms, neuronal NOS (nNOS) and endothelial NOS (eNOS) are constitutively expressed in myocytes [68]. Whereas eNOS expression and activity were shown to be normal in the dystrophic heart [69,70], nNOS expression [69] and activity [69,70] were significantly diminished, and the enzyme seemed to be mislocalized [71]. This would be consistent with the view that the presence of dystrophin is required for a proper expression and localization of nNOS. Indeed, in healthy cardiomyocytes, nNOS binds to dystrophin as well as syntrophin, and is part of the DAPC [72]. If nNOS is knocked out in animal models or is pharmacologically inhibited, currents through L-type Ca^2+^ channels are increased, and their inactivation is slowed [67,73,74]. Thus, both the enhanced I_Ca_ and the slowed channel inactivation observed in dystrophic cardiomyocytes (see above) could be explained by an abnormally decreased nNOS activity in the dystrophic cells. Direct proof for this hypothesis, however, is lacking as of yet. In addition, although we have been trying hard to reproduce the NO- or nNOS-mediated regulation of Ca_v_1.2 function in healthy mouse cardiomyocytes in our lab, we have failed so far (unpublished data). This is supported by the work from other groups, which contradicts the widely accepted view of L-type Ca^2+^ channel inhibition by NO via nNOS activity. Thus, cardiomyocytes from nNOS-/- mice exhibited normal I_Ca_ [75], and NO donors did not affect I_Ca_ in healthy rat myocytes [76]. Furthermore, diminished nNOS expression in mdx versus normal wild type hearts (see above) has been questioned [70,77]. In our opinion, further research is needed in order to validate or disproof the widely accepted view that nNOS functionally regulates the L-type Ca^2+^ channel in the heart. If nNOS, against the current view, does not significantly regulate this channel, its potential alteration in dystrophic cardiomyocytes can also not account for the L-type Ca^2+^ channel gain-of-function in these cells.

A further potential source for I_Ca_ abnormalities in dystrophic cardiomyocytes includes an altered regulation by reactive oxygen species (ROS). In particular, the Ca_v_1.2 alpha1 subunit is a target for direct redox modification during oxidative stress [78], and oxidizing agents result in an increase in the channel-mediated Ca^2+^ influx [79,80,81]. Consequently, increased ROS levels in the dystrophic heart [82] may help to explain the gain-of-function Ca^2+^ channel abnormalities in dystrophic cardiomyocytes.

Recently, it was shown that the expression of the nuclear pore protein Nup153 was significantly increased at protein level in the mdx hearts when compared with controls, and its overexpression in normal cardiomyocytes increased Ca_v_1.2 channel function [83]. This would also be in line with Ca^2+^ channel gain-of-function in dystrophic cardiomyocytes.

Besides the above-mentioned regulators as potential sources for abnormal Ca^2+^ channel function in dystrophin-deficient cardiomyocytes, numerous other known channel regulators, such as, for example protein kinase C (PKC), Ca^2+^/calmodulin-dependent protein kinase II (CaMKII), and A-kinase anchoring proteins (AKAPs) are further candidates as causative mechanisms or contributing factors. To the best of our knowledge, for none of these or other regulators, however, a conclusive link to dystrophic Ca^2+^ channel abnormalities has been established as yet.

Finally, because Ca_v_1.2 is an example of extreme splicing diversity [84], potential alterations in Ca^2+^ channel splice variants in the dystrophic heart, although currently unknown, should not be left out of consideration.

Despite increased I_Ca_ in dystrophin-deficient compared with wild type ventricular cardiomyocytes (see above), and the consequently increased Ca^2+^ influx during an AP, we and others [35,49] did not observe AP prolongation in these cells. This was probably due to the comparably small contribution of L-type I_Ca_ to the short AP in the murine heart [85]. Accordingly, the implementation of the experimentally observed enhancement in “dystrophic” Ca^2+^ conductance in a computer model of a human ventricular cardiomyocyte considerably prolonged the AP [49]. The ECG parameters most likely to be affected by Ca^2+^ channel abnormalities in dystrophic cardiomyocytes are the PQ and the QT intervals. The former parameter reflects the AV nodal conduction, whereas the latter parameter corresponds to AP duration, and is dominated by ventricular repolarization. In accordance with increased I_Ca_ in dystrophic cardiomyocytes, we and other authors have consistently found shortened PQ intervals in dystrophic mice (e.g., [49,56]). Short PQ intervals compared to control animals were also found in a canine model of DMD [86]. Regarding the QT interval, both normal (e.g., [34]) and prolonged (e.g., [27,56]) values compared to wild type mice were observed in dystrophic mice. In our study, mdx mice developed a small but significant QT interval prolongation only at an advanced age [49]. Taken together, Ca^2+^-dependent ECG parameters are altered in dystrophic mice, and should be considered a source for the emergence of cardiac arrhythmias, as described to occur in these animals [87,88].

Increased Ca^2+^ influx into the dystrophic cardiomyocyte during an AP may contribute to cellular Ca^2+^ overload [89]. Several authors have reported increased resting free intracellular Ca^2+^ concentrations in dystrophic cardiomyocytes [47,90,91], and there is evidence to suggest that excess intracellular Ca^2+^ is a key trigger of cell death and fibrosis in the course of dystrophic cardiomyopathy [8,9,10]. In our opinion, however, it is still unclear if, or to what extent L-type Ca^2+^ channel gain-of-function contributes to increased resting intracellular Ca^2+^ in dystrophic cardiomyocytes. Here, other factors such as, for example, altered TRP channel activity [30,31] and leaky ryanodine receptors [88] may provide major contributions.

Ca^2+^ influx into the cytosol triggers Ca^2+^-induced Ca^2+^-release from the SR via the ryanodine receptor. Thus, the enhanced Ca^2+^ influx into dystrophic cardiomyocytes during an AP may further alter the cells’ intracellular Ca^2+^ release, and consequently modulate the contractility. To investigate this, electrically evoked Ca^2+^ transients have been compared between wild type and mdx ventricular cardiomyocytes by several groups, with very contradictory results. Thus, the authors of [90] reported increased Ca^2+^ transient amplitudes in mdx cardiomyocytes, whereas others [48,77,88] found them to be normal or even diminished in mdx cells, respectively. The authors of [42] recently reported Ca^2+^ transient amplitudes in mdx cardiomyocytes to be either normal or increased when compared to wild type, and this was dependent on the age of the mice used. The latter report suggests that the different age ranges of mice used in the enumerated studies may explain at least part of their contradictory findings. In our study, electrically evoked Ca^2+^ transients in ventricular cardiomyocytes derived from aged mdx mice showed normal amplitudes when compared to wild type [52]. On the other hand, we found a significantly slowed decay of the Ca^2+^ signal triggered by electrical stimulation in “aged” mdx myocytes, which coincides with similar results in the literature (e.g., [77,90]). The latter finding can be explained by impaired Ca^2+^ removal from the cytosol after release in dystrophic cells [90]. The actually expected increase in Ca^2+^ transient amplitude due to enhanced L-type I_Ca_ in dystrophic cardiomyocytes (see above), however, cannot be derived from the as yet published literature.

Finally, an interesting new aspect in the context of L-type Ca^2+^ channel dysregulation in dystrophic cardiomyocytes was recently raised. In healthy myocytes, the L-type Ca^2+^ channel communicates with the mitochondria, and thereby regulates the cellular metabolic activity [44,50]. This communication occurs via the interaction of both the channel and mitochondria with the cytoskeleton, and involves a voltage-dependent anion channel (VDAC) in the outer mitochondrial membrane [44,92]. In the case of dystrophin deficiency, functional communication between the Ca^2+^ channel and mitochondrial VDAC is impaired, and this contributes to metabolic inhibition—a relevant feature of the dystrophic heart. Via this mechanism, L-type Ca^2+^ channel abnormalities are linked to metabolic dysfunction in dystrophic cardiomyopathy. The authors of [93] reported significantly diminished VDAC1 protein levels in mdx compared to healthy mouse hearts, whereas others [94] found a slight VDAC1 upregulation in dystrophin-deficient hearts using the mdx^4cv^ DMD mouse model.

### 2.3. Potassium Channels

In cardiomyocytes, voltage-dependent potassium (K^+^) channels are major determinants of the resting membrane potential and AP repolarization. In contrast to Na^+^ and Ca^2+^ channels (see above), very little is known about voltage-dependent K^+^ channels and potential abnormalities in their properties in the dystrophic heart. In an early study using the mdx mouse model [47], no significant difference was found in the voltage dependence and density of the transient outward K^+^ currents (I_to_) between the control and mdx ventricular cardiomyocytes. The authors of [53], using a canine model of DMD, reported significantly reduced I_to_ in dystrophic compared with control epicardial myocytes. The authors concluded that the reduction of I_to_ may alter the balance of inward and outward currents in the dystrophic myocardium, and thereby contribute to cardiac pathology. In our previous work, with a focus on the L-type Ca^2+^ channel [49], we found similar outward K^+^ currents in control and dystrophic ventricular cardiomyocytes derived from two DMD/BMD mouse models (mdx and mdx-utr). Recently, we have compared I_K1_ inward rectifier K^+^ currents in wild type and dystrophic (mdx and mdx-utr) ventricular cardiomyocytes [46]. The I_K1_ current, mainly carried by K_ir_2.1 channels [95], controls the resting membrane potential and the final phase of AP repolarization. Similar to Na_v_1.5 (see above), K_ir_2.1 is a member of the DAPC, and binds to syntrophin’s PDZ domain via its C-terminus [29,96]. Thus, we hypothesized that dystrophin deficiency might impair I_K1_ currents. Indeed, we found that I_K1_ was substantially diminished in the dystrophic compared with wild type cardiomyocytes [46]. The K_ir_2.1 protein levels in dystrophic compared to wild type ventricles were similar [46], or only slightly reduced [34]. This suggested that K_ir_2.1 channel expression is comparable in dystrophin-deficient and wild type cardiomyocytes. Furthermore, immunostaining experiments implied normal K_ir_2.1 localization within the T-tubular system in dystrophic cardiomyocytes [46]. These findings prompted us to suggest that another mechanism than the impaired K_ir_2.1 expression and/or localization must be responsible for the reduced I_K1_ currents in dystrophic cardiomyocytes. Here, channel inhibition by cytoplasmic regulatory factors is likely, and follow-up studies are needed in order to identify the responsible regulator(s). Finally, reduced I_K1_ currents due to dystrophin deficiency may provide an explanation for the low resting potentials occasionally measured in dystrophic cardiomyocytes [35,88]. They also represent a potential additional mechanism to cause arrhythmias in the dystrophic heart.

### 2.4. Ion Channel Abnormalities Prior to Dystrophic Cardiomyopathy Development

The described ion channel abnormalities in cardiomyocytes derived from dystrophin-deficient DMD/BMD mouse models may represent a primary effect of the dystrophin gene mutation and precede dilated cardiomyopathy development. As such, they could be regarded as a potential trigger of disease (see below). On the other hand, they may simply be a secondary effect generated by the cardiomyopathy. Thus, it is well known that heart failure leads to so-called “electrical remodeling”, including alterations of ion channel expression and function [97]. Lately, evidence for the view that channel abnormalities do exist already prior to cardiomyopathy development has been accumulating.

We could recently show that significant Na^+^ channel impairments (reduced current densities, slowed inactivation kinetics) are already present in dystrophin-deficient cardiomyocytes derived from one- to two-day-old neonatal mdx and/or mdx-utr mice [33]. As it is well established that cardiac abnormalities are lacking in these DMD/BMD mouse models, even in young adult animals (e.g., [40,98,99]), these Na^+^ channel defects in dystrophic neonatal cardiomyocytes can be considered primary effects of the dystrophin gene mutation, which precede cardiomyopathy development. Our auxiliary finding of reduced L-type Ca^2+^ channel inactivation in dystrophic neonatal cardiomyocytes [33], supported by the authors of [27], suggests that this is not a special feature of Na^+^ channels alone, but more generally holds true for other ion channels. To demonstrate the functional relevance of the observed Ca^2+^ channel abnormalities in dystrophic neonatal cardiomyocytes at the organ level, we performed ECG recordings on two-day-old neonatal mice. We found significantly shortened PQ-intervals in the dystrophic compared with wild type animals [49]. These findings, consistent with the impaired Ca^2+^ channel inactivation in dystrophic neonatal cardiomyocytes [27,33], suggested that atrioventricular (AV) nodal conduction is already accelerated in the dystrophic neonatal heart prior to cardiomyopathy development. Moreover, mitochondrial dysfunction due to impaired communication with the L-type Ca^2+^ channel is one of the first pathological alterations to occur in the mdx heart prior to the onset of any detectable cardiomyopathy [9,100].

Taken together, there are reports of “early” ion channel abnormalities in cardiomyocytes derived from dystrophin-deficient DMD/BMD mouse models. These are consistent with the concept of dystrophin (and DAPC) regulation of cardiac ion channel expression and function [33,49,101,102]. Importantly, ion channel abnormalities may be considered novel causes or underlying mechanisms of dystrophic cardiomyopathy genesis, as we have previously proposed [33]. Accordingly, in genetically engineered mice, dilated cardiomyopathy can be induced by the aimed alteration of cardiac ion channel expression and/or gating. For example, the reduced expression of Na_v_1.5 channels (a hallmark of dystrophic cardiomyocytes [34,35], see above) triggered dilated cardiomyopathy development [103].

## 3. Dystrophic Cardiac Ion Channel Abnormalities: Evidence from Human Studies

Although animal models of cardiomyopathy and heart failure are valuable tools to study disease mechanisms [104], the yielded findings on dystrophic animals may not always apply to human DMD/BMD patients. In particular, small animals, such as mice or rats, are often considered rather inappropriate models, because the physiology of their hearts is different to that of humans. For example, ion channel expression in adult ventricular cardiomyocytes from mice and humans is diverse, and this entails considerable differences in cellular AP shape (short AP duration and lack of distinct plateau phase in the mouse). Consequently, studies on ventricular cardiomyocytes from dystrophinopathy patients would be necessary in order to validate the data deduced from muscular dystrophy animal models. Because of the obvious difficulty in obtaining cardiomyocytes from human patients (and from appropriate healthy control individuals), there are no data on voltage-dependent ion channel abnormalities in native cardiomyocytes from dystrophinopathy patients, as yet. However, studies using cardiomyocytes derived from induced pluripotent stem cells (iPSCs) of dystrophinopathy patients begin to reveal first evidence. In addition, indirect evidence for abnormal ion channel function in the human dystrophic heart can be inferred from abnormal ECGs, impaired impulse conduction, and the occurrence of cardiac arrhythmias in patients.

L-type I_Ca_ densities, measured with the whole cell patch clamp technique, were significantly reduced in DMD iPSC-derived compared with control cardiomyocytes [105]. This is in contrast to the Ca^2+^ channel gain-of-function abnormalities consistently reported for dystrophic mouse ventricular cardiomyocytes (see above). As, to the best of our knowledge, the authors of [105] have been the only ones to compare I_Ca_ in normal and dystrophic human iPSC-derived cardiomyocytes, as yet, this single finding should not be over-interpreted.

Ca^2+^ handling was studied previously [106], and a difference was found between iPSC-derived DMD and healthy control cardiomyocytes, namely: the duration of recovery of the DMD Ca^2+^ transient triggered by electrical stimulation was significantly prolonged compared to the control. This is in line with similar findings in mouse models (e.g., [52,77,90]), and may be explained by impaired Ca^2+^ removal from the cytosol after release in dystrophic cells [90]. The authors of [105] reported elevated levels of resting Ca^2+^ concentration in DMD iPSC-derived cardiomyocytes, again in accordance with respective findings in mouse model studies [47,90,91].

Apart from iPSC-derived cardiomyocyte studies, there is indirect evidence for potentially abnormal voltage-dependent ion channel function in the human dystrophic heart, as follows: (i) like in dystrophic mouse ventricular cardiomyocytes, I_Na_ may be reduced in dystrophic human cardiomyocytes. Thus, ventricular conduction is impaired in DMD [107,108] and BMD [6] patients, and the QRS-interval in the ECG of patients with dilated cardiomyopathy is often prolonged (e.g., [109]); (ii) Like in dystrophic mouse ventricular cardiomyocytes, I_Ca_ may be increased in dystrophic human cardiomyocytes, because Ca^2+^-dependent ECG parameters in dystrophinopathy patients are altered in a respective manner, as follows: Patients typically show short PQ intervals [6,107,110,111], consistent with accelerated atrioventricular (AV) nodal conduction due to enhanced I_Ca_ in AV nodal cells, and in some cases also prolonged QT intervals [110,112,113]. A prolonged QT interval, however, is not a regular observation in dystrophinopathy patients, and may also arise via other mechanisms (i.e., abnormal K^+^ currents in dystrophic human ventricular cardiomyocytes). A further argument for potentially increased L-type I_Ca_ in dystrophic human cardiomyocytes can be found in the following recent study [83]: Nup153, a nuclear pore protein that has been shown to increase the Ca_v_1.2 channel expression and function in mouse cardiomyocytes, is up-regulated in the heart of DMD patients. Finally, the I_K_-dependent ECG parameters (i.e., QT interval and T-wave morphology) can also be abnormal in dystrophinopathy patients [110,112,113]. Here, a direct assignment to one or several particular K^+^ channels in the dystrophic heart, however, is currently impossible.

Taken together, the nature of the ECG abnormalities observed in dystrophinopathy patients, in comparison with the cellular ion channel and ECG impairments reported for mouse models of DMD/BMD (see above), suggest that the major ion channel abnormalities in dystrophic human and mouse hearts are similar, to a substantial extent. Furthermore, in analogy to the “early” ion channel abnormalities in cardiomyocytes from dystrophin-deficient DMD/BMD mouse models (see above), ECG abnormalities already occur in very young dystrophinopathy patients and precede the onset of cardiac dysfunction [20,114].

## 4. Limitations of the Experimental Studies

Functional ion channel data from native cardiomyocytes of dystrophinopathy patients (and appropriate healthy control individuals) are lacking. Thus, the actual existence of voltage-dependent sarcolemmal ion channel abnormalities in cardiomyocytes from DMD/BMD patients is currently unproven.

Studies using cardiomyocytes derived from iPSCs of dystrophinopathy patients currently suffer from issues like potentially different levels of cell maturity between control and DMD/BMD iPSC-derived cardiomyocytes, and/or genetic differences between cell donors (patients and healthy control individuals). This may impact the results of the electrophysiological recordings and lead to artificial findings.

The vast majority of the current knowledge about voltage-dependent ion channel abnormalities in the dystrophin-deficient heart has been gained from studies on the mdx mouse model. As already mentioned above, the physiology of the mouse heart is very different to that of the human heart. In addition, the mdx mice exhibit a rather mild cardiomyopathy with a later onset (e.g., [98]) when compared with dystrophinopathy patients. Therefore, the mdx mouse cannot be considered a fully appropriate animal model for DMD/BMD.

Finally, voltage-dependent ion channel activity depends on the mechanical strain put on a cardiomyocyte [115]. In the case of dystrophin deficiency, normal mechano-sensation is disrupted [116,117]. None of the isolated cardiomyocyte studies cited in the present review article have tested the effects of mechanical stress on the electrophysiological properties in normal and dystrophic cardiomyocytes. Dystrophic ion channel abnormalities under mechanical stress may be different to those observed under an unphysiological “unloaded” condition.

## 5. Conclusions, Clinical Implications, and Future Perspectives

The studies considered in the present review article clearly suggest the existence of significant voltage-dependent sarcolemmal ion channel abnormalities in the dystrophin-deficient heart. These are summarized in the cartoon displayed in Figure 1. A list of the occurring “dystrophic” changes in the ion channel properties at the level of protein expression and function is given in sections A and B of Table 1, respectively.

Dystrophic ventricular cardiomyocytes show considerable Na^+^ channel loss-of-function and L-type Ca^2+^ channel gain-of-function. In addition, the functional communication between the Ca^2+^ channel and mitochondria is impaired [44], which provides a link to metabolic dysfunction in dystrophic cardiomyopathy. Furthermore, the activity of some K^+^ channels may be reduced in dystrophic myocytes. Ion channel abnormalities occur prior to cardiomyopathy development in the dystrophic heart, and may thus represent a primary effect of the dystrophin gene mutation. Channel abnormalities in dystrophic cardiomyocytes can explain abnormal ECG parameters observed in dystrophic animals and dystrophinopathy patients, and represent a relevant source for the emergence of cardiac arrhythmias. Dystrophic Na^+^ channel impairments may be considered as a major cause of the cardiac conduction defects observed in DMD/BMD patients. L-type Ca^2+^ channel gain-of-function in dystrophic cardiomyocytes, on the other hand, may give rise to Ca^2+^-dependent arrhythmias. It is interesting in this context, that similar Ca^2+^ channel abnormalities (larger currents and impaired inactivation) were shown to underlie the serious cardiac arrhythmias observed in Timothy syndrome, a rare multisystem disorder caused by Ca_v_1.2 channel mutations [118,119,120]. Furthermore, as ion channel impairments and associated mitochondrial dysfunction in cardiomyocytes precede the onset of cardiomyopathy development in the dystrophic heart, they may well play a role in triggering the disease. Accordingly, both reduced I_Na_ [103] and increased I_Ca_ [121] in cardiomyocytes initiated cardiomyopathy development in mouse model studies. Consequently, ion channel modulation may not only represent a tool for arrhythmia management in dystrophinopathy patients, but emerges as a promising candidate for the development of new treatment strategies to prevent dystrophic cardiomyopathy genesis and to stop the progression of the disease. Studies with animal models of muscular dystrophy are required to thoroughly test this hypothesis, as the potential basis for subsequent clinical trials.

In order to gain deeper insights, prospective studies should focus on more appropriate DMD/BMD animal models for the cardiac disease phenotype other than dystrophic mice. Here, a just recently described rabbit model of muscular dystrophy [122] is of particular interest, as the properties of the rabbit heart more closely resemble those of human hearts [123]. For example, in contrast to the ventricular cardiomyocytes from adult mice, rabbit myocytes show a distinct plateau phase in their AP, and express K_v_11.1 (hERG) and K_v_7.1 (KCNQ1) channels, the major determinants of AP repolarization in human ventricular cardiomyocytes (e.g., [124]). Because of their absence in adult mouse ventricular myocytes, the potential abnormalities of these important K^+^ channels in the dystrophic heart have not been studied so far.

A gain of further knowledge is also expected from future studies with human DMD/BMD patient iPSC-derived cardiomyocytes. However, this will depend on methodological improvements yet to be achieved: e.g., the gain of truly mature iPSC-derived cardiomyocytes, or the avoidance of the generation of strongly heterogeneous myocyte populations, which is a typical shortcoming of current approaches to obtain iPSC-derived cardiomyocytes (e.g., [125,126]).

Finally, the current knowledge regarding the abnormalities in voltage-dependent sarcolemmal ion channel functional properties in the dystrophic heart is almost exclusively based on studies on ventricular cardiomyocytes. Prospective studies should also consider myocytes from other heart regions, such as AV-nodal cells and cells from the central ventricular conduction system of the heart. This would certainly help to better understand the emergence of cardiac arrhythmias in the dystrophic heart.

## Figures and Tables

**Table 1:** Dystrophin deficiency—induced changes in the properties of cardiac ion channels at the level of protein expression (**A**) and channel function (**B**). Arrow heads pointing towards the bottom indicate a down-regulation of channel protein expression (**A**), or a channel loss-of-function (**B**). “=” represents no change in channel expression (**A**) or function (**B**). Arrow heads pointing towards the top indicate an up-regulation of the channel protein expression (**A**), or channel gain-of-function (**B**). na (not available) implies that it is currently unknown if dystrophin deficiency induces any changes in the expression of the respective channel protein. I_Na_—Na^2+^ current; I_CaL_—L-type Ca^2+^ current; I_CaT_—T-type Ca^2+^ current; I_K1_—inward rectifier K^+^ current; I_to_—transient outward K^+^ current; I_K,slow_—ultra-rapid delayed rectifying and slowly inactivating K^+^ current; mdx—dystrophin-deficient, classical dystrophinopathy mouse model; mdx^5cv^—mdx strain with a different point mutation; mdx-utr—both dystrophin- and utrophin-deficient, more severe disease phenotype. The channel or channel subunit names are given according to IUPHAR (International Union of Basic and Clinical Pharmacology; http://www.guidetopharmacology.org) nomenclature.

(A) Protein Data					
Current	Ion Channel	Gene Name	Change	DMD Model	Reference
**I_Na_**	Nav1.5	Scn5a	↓	mdx, mdx-utr	[35]
			↓	mdx	[41]
			↓	mdx^5cv^	[34]
			↓	mdx^5cv^	[36]
	β1 subunit	Scn1b	na		
**I_CaL_**	Cav1.2	Cacna1c	=	mdx^5cv^	[34]
			=	mdx	[42]
			=	mdx	[43]
			=	mdx	[44]
	β2 subunit	Cacnb2	=	mdx	[42]
	α2δ1 subunit	Cacna2d1	=	mdx	[45]
**I_CaT_**	Cav3.1	Cacna1g	na		
	Cav3.2	Cacna1h	na		
**I_K1_**	Kir2.1	Kcnj2	↓	mdx^5cv^	[34]
			=	mdx, mdx-utr	[46]
**I_to_**	Kv4.2	Kcnd2	na		
	Kv4.3	Kcnd3	na		
	Kv1.4	Kcna4	na		
**I_K,slow_**	Kv1.5	Kcna5	na		
	Kv2.1	Kcnb1	na		
**(B) Functional Data**					
**Current**	**Ion Channel**	**Gene Name**	**Change**	**DMD Model**	**Reference**
**I_Na_**	Nav1.5	Scn5a	↓	mdx, mdx-utr	[35]
			↓	mdx^5cv^	[34]
			↓	mdx, mdx-utr	[33]
			↓	mdx^5cv^	[36]
**I_CaL_**	Cav1.2	Cacna1c	=	mdx	[47]
			=	mdx	[48]
			↑	mdx, mdx-utr	[33]
			↑	mdx, mdx-utr	[49]
			↑	mdx	[42]
			↑	mdx	[27]
			↑	mdx	[50]
			↑	mdx	[44]
			↑	mdx	[51]
**I_CaT_**	Cav3.1, Cav3.2	Cacna1g, Cacna1h	=	mdx	[52]
**I_K1_**	Kir2.1	Kcnj2	↓	mdx	[46]
**I_to_**	Kv4.2, Kv4.3, Kv1.4	Kcnd2, Kcnd3, Kcna4	=	mdx	[47]
			↓	xmd dog	[53]
**I_K,slow_**	Kv1.5, Kv2.1	Kcna5, Kcnb1	=	mdx, mdx-utr	[49]
